# The Foreign Journals: Animal Chemistry and Pharmacy

**Published:** 1842-04

**Authors:** 


### ANIMAL CHEMISTRY AND PHARMACY.

*Remarks on the Memoir of Dr. Dupasquier, on the Protioduret of Iron, with new formulæ for the preparation of this remedy.* By M. F. Boudet.

M. Boudet objects to M. Dupasquier's formulæ, that, according to his method, the protioduret of iron always requires extemporaneous preparation, and that the quantities of the medicine contained in the different preparations of it are very small and fractional, so as to occasion considerable difficulty in calculating their doses. He therefore suggests the following as an officinal preparation, which contains one part in ten of protioduret of iron:

| | | | Parts. |
|---|---|---|---|
| Take of | pure iodine, | by weight | . . 8½ |
| ,, | Iron filings | ,, | . . 4 |
| ,, | Distilled water | ,, | . . 40 |
| ,, | Best white sugar | ,, | . . 55 |
| ,, | Gub arabic, in powder | ,, | . . 8 = 115½ |

Mix the iodine and thirty parts of water together in a glass flask, add the iron filings gradually, shaking the mixture all the time, heat it slightly until the liquid has become nearly colourless; then filter, letting the filtered liquid drop into an iron capsule containing the sugar coarsely powdered. Wash the filter with the ten remaining parts of water, and dissolve in it the gum ; pour this solution into the capsule, and expose the whole to heat till reduced to 100 parts, each of which will contain a tenth part by weight of pure protioduret of iron.

This solution may be preserved, without fear of decomposition, in a well-stopped bottle.

Prescriptions are given for various preparations which may be made from it. It will keep without changing for twenty-four hours in a mixture, provided that contain a tenth of its weight of syrup.

Syrup of protiodide of iron.

| | Parts. |
|---|---|
| Officinal solution of protiodide of iron . . . . | 20 |
| Syrup of gum . . . . . . . . . . . . . . | 220 |
| Syrup of orange flower . . . . . . . . . . | 60 = 300 |

[The above formulæ will, we are sure, be welcome to all who are in the practice of employing this valuable preparation, the only objection to which has been the readiness with which it becomes decomposed.]

*Bulletin Gén. de Thérapeutique. Sept.* 30, 1841.

